# Next Generation of Weight Management Medications: Implications for Diabetes and CVD Risk

**DOI:** 10.1007/s11886-015-0590-z

**Published:** 2015-04-18

**Authors:** S. Wharton, K. J. Serodio

**Affiliations:** 1 Weight Management and Diabetes Management, The Wharton Medical Clinic, 414 Victoria Ave N Suite 14, Hamilton, ON L8L 5G8 Canada; 2 Department of Health and Kinesiology, York University, 4700 Keele St, Toronto, ON M3J 1P3 Canada

**Keywords:** Weight management, Medications, Cardiometabolic risks

## Abstract

Since the 1980s, the prevalence of obesity has almost doubled worldwide. Treatments for obesity include lifestyle modification, medications and surgery. Newer anti-obesity medications have been shown to be effective at inducing initial weight management in addition to successful long-term weight maintenance. Historically, weight management medications have been associated with public safety concerns that have resulted in the majority being withdrawn from the market or never receiving medicinal authorization. Recently, several countries have approved some newer generation weight management medications which may be beneficial to combat obesity. These medications have varying effects on cardiometabolic parameters, both positive and potentially negative. This review will outline the mechanisms of action of these medications and their implications for both diabetes and cardiovascular risks.

## Introduction

### Overview and Prevalence

Since the 1980s, the prevalence of obesity has almost doubled worldwide, with over 500 million men and women being classified as obese [1]. Obesity is a condition characterized by excessive body fat and is often associated with pathophysiological complications to many endogenous systems including metabolic, cardiovascular, endocrine, central nervous and more [2]. Furthermore, obesity is a complex, chronic condition that is resistant to most standard treatments [3]. It is well established that a modest weight loss of 5 to 10 % can decrease many weight-related cardiometabolic risk factors such as elevated glycaemic markers, abnormal blood lipids, increased uric acid concentrations and hypertension [4, 5]. Unfortunately, most patients have difficulty maintaining even this small amount of weight loss likely due to the activation of weight-preservation hormones and pathological changes to biological systems [3].

Weight management treatments focused on lifestyle intervention are generally linked with short-term weight loss followed by weight regain within 2 years [4, 6]. Nonetheless, standard of treatment recommended for weight management is still lifestyle modification, with other measures such as pharmacological intervention and bariatric surgery acting as secondary and tertiary options [7]. Medications will be very useful to bridge the gap between lifestyle modifications and surgery and have been shown to increase the success of long-term weight maintenance. In the past 10 years, we have seen the development of several pharmacological agents for weight management [6]. These medications have beneficial effects on metabolic conditions such as diabetes, yet their effects on cardiovascular disease are still unclear. This review will outline the new generation of weight management medications and the implications for both diabetes and CVD.

### Pharmacotherapy Availability and Regulation

Although bariatric surgery has been shown to be a very effective weight management option, it is only offered to patients with a BMI ≥40 kg m^2^ or a BMI ≥35 kg m^2^ with one or more obesity-related comorbidity [6]. In contrast, weight management medications can be more widely prescribed to anyone with a BMI ≥30 kg m^2^ or those who have a BMI >27 kg m^2^ with at least one obesity-related comorbidity [8, 9]. Thus, pharmacotherapy treatment has the potential to be much more readily available to a greater proportion of persons with obesity.

Currently, there are three categories of anti-obesity drugs, including (1) central nervous system modifiers, (2) endocannabinoid inhibitors and (3) fat absorption inhibitors [4]. Prior to medicinal licencing and commercialization, medications must meet the guidelines by drug enforcement agencies such as Health Canada, the American Food and Drug Administration (FDA) and the European Medicines Agency (EMA). In past years, these agencies have limited the availability of anti-obesity medications due to public health concerns that frequently overshadow the relatively modest results received from pharmacotherapeutic treatment [4, 5].

### FDA Requirements for Approval of Anti-obesity Drugs

Anti-obesity medication approval by the FDA requires a 24-month study demonstrating a minimum 5 % placebo-subtracted weight loss and that at least 35 % of the treatment group, or a significantly greater proportion of treatment subjects, maintain ≥5 % weight loss from their initial body weight [5, 10]. Similarly, the EMA guidelines require a ≥5 % placebo-subtracted weight loss from baseline and recommend that trials at least 6 months in duration with one pivotal trial lasting ≥12 months [11]. Medications are also expected to improve the body composition, health risk profile and health-related quality of life (QoL) of patients as otherwise weight management would merely be for aesthetic purposes [10, 11]. FDA and EMA medication approvals are usually accompanied by the requirement of post-marketing adverse event monitoring and cardiovascular risk research studies [6].

### History and Cardiometabolic Consequences of Withdrawn Medications

Since the 1930s, the majority of anti-obesity medications that were once approved have been withdrawn from the market due to adverse effects and threat to public health [3, 7]. The side effect of these medications included disturbances to the psychological, central nervous and cardiovascular systems [3, 6, 7]. The once very popular sympathomimetic compounds ephedrine-alkaloids were withdrawn due to adverse cardiovascular events, including arrhythmias and sudden cardiac death [12, 13]. Rimonabant (Acomplia®), a selective cannabinoid (CB1) receptor inhibitor effective at decreasing appetite, was also withdrawn from the market due to doubling the risk of psychiatric disorders, in particular depression [14, 15]. Fenfluramine-phentermine (Fen-Phen), a serotonin (5HT-_2b_) receptor activator with sympathomimetic properties and anorectic actions, was removed from the market in 1997 due to valvular heart disease and pulmonary hypertension [16–18]. Similarly, sibutramine (Meridia®) had sympathomimetic properties, inhibiting serotonin 5-HT reuptake, which helped to promote satiety, was also withdrawn in 2010 given its propensity to increase blood pressure, myocardial infarction and stroke risk, primarily in patients with existing CVD [18, 19]. The tendency for increased risk of cardiac events with the use of older anti-obesogenics is well established while the cardiometabolic effects of newer generation anti-obesogenics is still being investigated.

## Weight Management Medications

### Orlistat (Xenical®, Alli®)

**Fig. 1:**
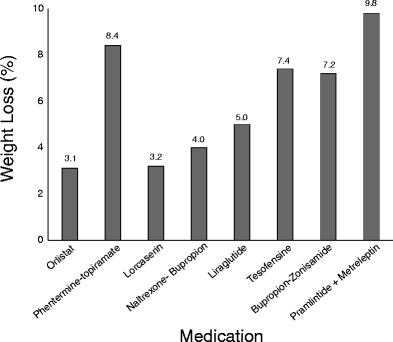
Average placebo-subtracted weight loss (%)

Orlistat is a pancreatic lipase inhibitor that decreases the absorption of dietary fats [4, 20]. Long-term medicinal treatment is associated with a dose-dependent 2.5 to 3.4 kg, 3.1 %, placebo-subtracted weight loss (Fig. 1) [20–22].

Cardiometabolic implications: Cardiometabolic risk factors such as total cholesterol (TC), low-density lipoprotein (LDL) cholesterol as well as systolic and diastolic blood pressure (BP) have all been noted to improve with long-term use (Table 1) [15, 21–23]. However, there have been no hard endpoint cardiovascular disease reductions with this medication, and long-term (≥1 year) patient discontinuation rates are high at approximately 90 % due to it being commonly linked with the malabsorption of fat-soluble vitamins, abdominal bloating, gastric pain and unpleasant steatorrhoea [10, 20].

**Table 1:** Changes to Cardiometabolic Markers

	Lipids				Vitals			Glycemic		
Medication	TC (%)	LDL (%)	HDL (%)	TG (%)	SBP (mmHg)	DBP (mmHg)	HR (bpm)	FBG (mM)	Insulin (pmol/L)	HbA1c (%)
Orlistat	-7.9-8.7 [22, 23]	-12.8-16.3 [22, 23]			-1.8-4.9 [15, 21, 22]	-1.6-2.6 [15, 21, 22]		-0.8 [15]	-32.0 [22]	
Phentermine-topiramate	-6.0-6.3 [27, 30]	-6.9-8.4 [27, 30]	+3.5-11.9 [27, 30, 31]	-5.2-13.7 [27, 30, 31]	-2.9-5.6 [27, 30]	-1.5-3.8 [27, 30]	+1.7 [30]	-0.03-0.07 [27, 30, 31]	-27.6 - 31.2 [30, 31]	-0.1- 0.2 [30]
Lorcaserin	-0.9 [34]	-1.2* [34]	+3.7-5.2 [33, 36]	-4.3-6.2 [34, 36]	-1.4 [34]	-1.1 [34]	-2.0 [33, 34]	-0.04-1.5 [33, 34]	-20.0 [34]	-0.04-0.9 [33, 34]
Naltrexone- Bupropion		-5.1 [39]	+6.5-9.4 [39–42]	-9.8-16.6 [39–42]	+1.1-1.8* [39, 40]	+0.9 * [40]	+1.0-2.5 [39, 40]	-0.18 [40]	-11.4-28.0 % [39, 40, 42]	-0.6 [41]
Liraglutide	-4.0 [44]	-13.0 [44]	+3.0 [44]	-14.0 [44]	-2.6-2.7 * [44, 51]			-0.4* [51]	-13.3 * [51]	-0.3* [51]
Tesofensine	-0.35*mM [58]	-0.25* mM [58]		0.38-0.39* mM [58]	+5.5* [58]	+4.3* [58]	+4.3-8.1* [58]		-17.8* [58]	-0.12* [58]
Bupropion-Zonisamide	Data not released.									
Pramlintide- Metreleptin	-9.0 [63]	-8.0 [63]		-8.0 [63]				-0.22 [63]	-16.8 [63]	

TC, total cholesterol; LDL, low-density lipoprotein; HDL, high-density lipoprotein; TG, triglycerides; SBP, systolic blood pressure; DBP, diastolic blood pressure; HR, heart rate; FBG, fasting blood glucose; Insulin, fasting insulin; HbA1c, glycated hemoglobin; mM, millimolar per litre, pmol/L, picomole per litre

Units stated in row #2 unless specified otherwise within the cell

*In comparison to placebo

Diabetic implications: The Xendos trial was one of the first studies with a weight management medication designed to demonstrate prevention of progression to diabetes in pre-diabetic patients [22]. This was a positive trial demonstrating a 37.3 % decrease in progression to diabetes [22]. The American Heart Association (AHA), American College of Cardiology (ACC), Endocrine Society and the Canadian Diabetes Association (CDA) guidelines all suggest orlistat as an effective therapy to assist in optimizing glycaemic markers and/or manage weight in patients with and without diabetes [24–26].

### Phentermine-Topiramate (Qsymia®, Qnexa®, Qsiva®)

Phentermine-topiramate controlled-release compounds help to suppress appetite [3]. Phentermine has been used for weight management in the USA for decades, but is rarely used in Canada and is off the market in Europe [6, 27]. Phentermine suppresses appetite through amphetamine effects [3]. Topiramate is indicated as an anti-epileptic medication that has also been associated with weight loss [3, 18, 27, 28•]. Topiramate’s mechanism for weight management is unclear [3, 18, 27]. Yet, both increased resting energy expenditure and anorectic properties may be causative [3, 26, 27]. The combination medication uses lower doses of each medication resulting more mild side effects than would be seen at usual doses [18]. Qsymia® was recently approved in the USA in 2012 but is yet to be approved by the Health Canada and EMA [3, 29]. Phentermine-topiramate FDA approval was primarily based on four studies that lasted between 24 and 56 weeks [7]. The EQUATE, EQUIP, CONQUER and SEQUEL trials reported placebo-subtracted weight losses of approximately 7.4 to 10.8 kg or between 7.5 and 9.3 % (Fig. 1) [3, 27, 30, 31]. Mild adverse events commonly linked with phentermine-topiramate include paresthesia, palpitations and elevated heart rate, while serious harms include an increased probability of cognitive deficits, metabolic acidosis and increased risk of birth defects [6, 20, 28•].

Cardiac implications: In the pivotal trials, phentermine-topiramate use was associated with improvements in surrogate cardiac markers including TC, LDL, high-density lipoprotein (HDL), triglycerides (TGs), BP, inflammatory markers and a reduction in the number of hypertensive medications patients required [27, 30, 31]. Conversely, phentermine-topiramate-treated subjects showed an elevated heart rate of 1.2 to 1.7 beats per minutes (bpm), in comparison to placebo subjects (Table 1) [27, 30, 31]. When submitting for FDA approval, preliminary major adverse cardiovascular events (MACE) data was reviewed and it was determined that there was no significant difference in the risk of cardiovascular death, non-fatal myocardial infarction and non-fatal stroke between phentermine-topiramate and placebo [28•]. That being said, cardiac safety analysis was not the primary goal of past studies and many were underpowered [28•]. The Endocrine Society’s Pharmacological Management of Obesity guidelines specify that, due to their sympathomimetic properties, phentermine-containing compounds should be not be prescribed to patients with a history of heart disease and uncontrolled hypertension until more detailed cardiac safety data is obtained [26]. The promise of a future cardiovascular event-specific trial called Aqclaim was a condition of FDA approval and will be beneficial in assisting both Health Canada and the EMA in making a firm decision on the safety of phentermine-topiramate therapy [28•, 32].

Diabetes implications: Phentermine-topiramate use is associated with improvements to numerous glycaemic markers such FBG, fasting insulin, glycated haemoglobin (HbA1c) and homeostatic model assessment of insulin resistance (HOMA-IR) (Table 1) [27, 30, 31]. Diabetic and pre-diabetic patients using phentermine-topiramate not only achieved clinically significant weight loss but also required fewer additional anti-diabetic drugs when compared against non-users [30]. Over a 2-year trial period, the significant weight loss associated with phentermine-topiramate use in non-diabetic patients slowed the progression of type 2 diabetes by 54 % [31].

### Lorcaserin (Belviq®)

Another newly approved medication by the FDA, in 2012, is Belviq®. Lorcaserin is a selective serotonin 2c (5HT-_2c_) receptor activator that promotes appetite suppression [7]. Lorcaserin is theorized to reduce energy intake through influencing the hypothalamic pro-opiomelanocortin (POMC) neurons within the central nervous system [7, 33, 34]. Health Canada approval is still pending, and EMA authorization was denied given the unexplained increase in various cancers seen in rodent studies and the potential risk for valvular pathology, although neither have been seen in human studies to date [3, 6, 35]. Three trials are commonly referenced when assessing the long-term effects of lorcaserin, BLOOM, BLOSSOM AND BLOOM-DM [33, 34, 36]. On average, a 3.2 kg, or 3.2 %, placebo-subtracted weight loss is expected with long-term (≥1-year) use, whereas shorter programmes between 8 and 12 weeks promote placebo-subtracted weight losses of 1.6 to 2.9 kg (Fig. 1) [7, 33, 34, 36]. Within the trials, the statistically significant placebo-subtracted percent weight loss from baseline ranged between 3.0 and 3.6 %, which is below the desired 5 % [33, 34, 36]. Nevertheless, approximately double the number of participants was able to attain a ≥5 % statistically significant weight loss in comparison to placebo (Table 2) [33, 34, 36]. Mild adverse events associated with lorcaserin use include headache, dizziness and nausea [3, 7].

**Table 2:** Proportion of participants expected to lose ≥5 %

Medication	Intervention	Placebo	Difference
Orlistat			
Torgerson 2004	72.8	45.1	27.7
Phentermine-topiramate			
Allison 2012	66.7	17.3	49.4
Gadde 2011	70.0	21.0	49.0
Garvey 2012	79.3	30.0	49.3
Average	72.0	22.8	49.2
Lorcaserin			
Smith 2010	47.5	20.3	27.2
Fidler 2011	47.2	25.0	22.2
O’neil 2012	37.5	16.1	21.4
Average	44.07	20.5	23.6
Naltrexone-bupropion			
Wadden 2011	66.4	42.5	23.9
Greenway 2010	48.0	16.0	32.0
Apovian 2013	50.5	17.1	33.4
Hollander 2013	44.5	18.9	25.6
Average	52.4	23.6	28.7
Liraglutide			
Astrup 2009	76.1	29.6	46.5
Wadden 2013	50.5	21.8	28.7
Average	63.3	25.7	37.6
Tesofensine			
Astrup 2008	91.0	29.0	62.0
Bupropion-zonisamide			
Orexigen 2014	82.6	18.9	63.7
Pramlintide-metreleptin			
Ravussin 2009	89.0	–^a^	–^a^

^a^Not placebo controlled

Cardiac implications: With long-term, twice daily, lorcaserin treatment TC, LDL, HDL, TGs, BP and heart rate (HR) all significantly improved (Table 1) [33, 34, 36]. As a result of the valvular complications associated with previous selective serotonin receptor activators, lorcaserin trials are obligated to include echocardiograms which to date have not documented any differences in valvular function [3, 6, 36]. The Endocrine Society recommends its use in patients with a history of heart disease over medications that have sympathomimetic effects [26]. Although lorcaserin treatment has been associated with a number of beneficial cardiometabolic improvements, the majority of trials have been of homogeneous populations and too underpowered to statistically test for echocardiographic safety [3, 33, 34, 36]. So, future research will be required before these findings can be generalized across ethnicities and gender. The FDA-requested cardiovascular safety trial for lorcaserin is called Camellia [37]. The 5-year multicenter TIMI study started recruiting in late 2013 and will include approximately 12,000 patients in 14 countries around the world [37].

Diabetes implications: For both diabetic and non-diabetic patients, lorcaserin treatment resulted in improvements in numerous glycaemic markers such FBG, fasting insulin, HbA1c and HOMA-IR (Table 1) [33, 34]. Lorcaserin-treated patients were also significantly less likely to have to increase their anti-diabetic medications when compared against non-users over a 1-year period [33]. The positive glycaemic influence of lorcaserin is likely weight dependent given that after 2 years of treatment some weight regain occurred, which consequently removed the beneficial effects of lorcaserin on glucose and insulin concentrations [6, 34].

### Naltrexone-Bupropion (Contrave®)

In the fall of 2014, FDA approval was given to an anorectic combination medication, of naltrexone and bupropion, called Contrave® [38•]. Naltrexone and bupropion are both used in the treatment of addiction [39, 40]. Mechanistically, the bupropion component of this combination therapy is thought to stimulate POMC neurons while naltrexone inhibits the opioid-mediated area of the POMC system [40]. Additionally, both drugs are believed to influence the mesolimbic dopaminergic reward system [41]. Six months of naltrexone-bupropion combination therapy is associated with a 3.8 to 4.9 kg, or 3.2 to 4.8 %, placebo-subtracted weight loss and 23 to 33 % more patients attaining a ≥5 % weight loss (Fig. 1 and Table 2) [3, 39–42]. Common minor adverse reactions associated with Contrave use include nausea, bowel/digestive upset, insomnia and headaches [38•, 40]. Some major adverse reactions include increased risk of neuropsychiatric events and increased risk of seizures [38•].

Cardiometabolic implications: Improvements to QoL and several cardiometabolic risk factors such as LDL, HDL and TGs have been linked with naltrexone-bupropion treatment [39–42]. Unfortunately, in comparison to placebo, naltrexone-bupropion use has been linked with a significantly smaller decrease in BP by 0.9 to 1.8 mmHg and elevated heart rate (Table 1) [39, 40]. Based on the recommendations from the Endocrine Society to avoid agents with sympathomimetic properties, naltrexone-bupropion should likely not be used as a first-line medication for patients with a history of cardiac dysfunction [26]. Electrocardiographic findings after more than 1 year of medicinal treatment did not find any differences between treatment and placebo [40]. Yet, the long-term consequences of the aforementioned increases in BP and HR with Contrave use are ambiguous so the FDA stipulated that additional cardiovascular safety outcomes are required. Thus, a long-term cardiovascular safety trial called *‘the Light Study’* is under way and should be completed by mid-2017 [3].

Diabetic implications: In non-diabetic patients, naltrexone-bupropion has been linked to a positive influence on FBG, fasting insulin, HbA1c and HOMA-IR without significantly increasing the prevalence of hypoglycaemic events [39–42]. Naltrexone-bupropion use in diabetic patients demonstrated beneficial effects on HbA1c levels and HbA1c goal attainment which ultimately resulted in significantly fewer participants requiring an increase in diabetic medications over 56 weeks of treatment (Table 1) [41]. It should be noted that the naltrexone-bupropion trials lacked heterogeneity as the vast majority of studies included white women without considerable co-morbidities (diabetes, cardiovascular disease) [39, 40, 42].

### Liraglutide (Saxenda®)

GLP-1R agonists, such as liraglutide, enhance insulin sensitivity, suppress appetite and delay gastric emptying [43]. Liraglutide works to stimulate the release of insulin from beta cells and suppresses glucagon secretion from alpha cells when blood glucose levels are elevated [44]. In 2009 and 2010, the FDA, EMA and Health Canada approved the use of liraglutide 0.6 to 1.8 mg for the treatment of type 2 diabetes [45, 46]. In December 2014 the FDA approved the use of liraglutide 3.0 mg for the treatment of obesity, shortly followed by Health Canada’s approval in February 2015 [47••, 48]. The trade name for liraglutide 3.0 mg is Saxenda® [47••]. The four trials frequently referenced when assessing the effects of liraglutide 3.0 mg are the SCALE, SCALE-Maintenance, SCALE-OSA and SCALE-Diabetes trials [49]. Liraglutide 3.0 mg-treated patients generally achieved a 4.4 to 5.9 kg or 3.9 to 6.1 % greater weight loss than placebo groups (Fig. 1) [44, 50, 51••]. Moreover, liraglutide 3.0 mg resulted in 29 to 47 % more of the liraglutide participants attaining a ≥5 % weight loss when compared against placebo participants and 32.5 % more being able to maintain significant weight loss after a lifestyle intervention (Table 2) [50, 51••]. Liraglutide was not only more effective at weight loss maintenance but also stimulated an additional 6.0 % greater weight loss than control groups in 56 weeks of medicinal treatment post-lifestyle intervention [51••]. Gastrointestinal upset such as nausea, vomiting and diarrhoea are the most frequent adverse events associated with liraglutide use [44, 50, 51••].

Cardiometabolic implications: The cardiovascular improvements associated with liraglutide 3.0 mg use include decreased TC, LDL, HDL, TGs and SBP (Table 1) [44, 51••]. The cardioprotective effectives of liraglutide in lower doses (1.2/1.8 mg, etc.) have been consistently demonstrated [43, 52–56]. These results must be taken with a degree of scepticism given that long-term cardiometabolic safety information at the 3.0-mg dosage proposed for weight management is not currently available. More informative cardiovascular safety data is expected by mid-2016 with the completion of the *“Liraglutide Effect and Action in Diabetes: Evaluation of Cardiovascular Outcome Results (LEADER)”* trial [56].

Diabetes implications: The SCALE-Maintenance trial looked at the influence of liraglutide 3.0 mg throughout a 56-week intervention, and as expected, liraglutide users had significantly improved FBG, fasting insulin and HbA1c measurements when compared against placebo participants (Table 1) [51••]. Furthermore, this trial noted that fewer liraglutide 3.0 mg users had to withdraw from their intervention due to type 2 diabetes onset when compared against non-users [51••]. Given that GLP-1R agonists are associated with not only successful weight loss but also glycaemic control, the Endocrine Society has suggested that they can be used as a first-line agent or an add on to other therapies for patients with diabetes [26]. To date, results indicate that liraglutide is effective at decreasing the weight and glycaemic markers of overweight and obese diabetic patients without substantially increasing the risk of hypoglycaemic events [44].

## Pipeline Medications

### Tesofensine

Originally produced as an Alzheimer’s disease treatment, tesofensine inhibits norepinephrine, serotonin and dopamine reuptake [10]. It was not found to improve Alzheimer’s disease but did result in substantial weight loss in a considerable number of patients [10]. Tesofensine was soon found to have anorectic properties in addition to the potential to increase energy expenditure [10, 57, 58]. Consequently, weight management trails were carried out, and they documented dose-dependent weight losses of 4.5 to 10.6 kg, or 4.4 to 10.4 %, of participants’ initial body weight with 59 to 91 % of patients achieving a ≥5 % weight loss (Fig. 1 and Table 2) [58]. Adverse events associated with its use include dry mouth, nausea and constipation [58].

Cardiometabolic implications: After 24 weeks, at various doses, of tesofensine use, TC, LDL and TG levels improved in non-diabetic participants [58]. Regrettably, increases to hemodynamic measurements such as BP and HR also occurred with tesofensine, and a small amount of weight regain occurred after medication discontinuation (Table 1) [58].

Diabetes implications: Tesofensine has been found to have beneficial effects on plasma insulin and HbA1c concentrations (Table 1) [58]. However, there is very limited data to substantiate these findings as the majority of trials have been completed on rodents, are not published in English or are methodologically flawed [57].

### Bupropion-Zonisamide Slow-Release Compound (Empatic®)

A bupropion-zonisamide slow-release compound, Empatic®, is undergoing FDA phase III clinical development [59, 60]. Bupropion is an antidepressant and smoking cessation drug that stimulates norepinephrine and dopamine activity [61]. Zonisamide is indicated as an anti-epileptic drug with mechanisms that influence serotonin and dopamine [61]. Together, bupropion-zonisamide has the potential to influence all three major neurotransmitters that regulate energy expenditure and appetite [61]. Previous trials document bupropion-zonisamide use to result in a 7.2 % placebo-subtracted reduction in weight from baseline with 63 % more of the medicinal users losing ≥5 % of their initial body weight (Fig. 1 and Table 2) [10, 60, 62••]. Bupropion and zonisamide have been combined not only due to their anorectic properties but also due to the offsetting adverse effect profile of each medication [61]. Bupropion monotherapy is characterized by psychomotor agitation and insomnia while zonisamide use is associated with somnolence, psychomotor inhibition, teratogenicity and depression [61]. In comparison to monotherapy, bupropion-zonisamide use has a more mild adverse reaction profile inclusive of headaches, nausea and insomnia [4, 60].

Cardiovascular implications: Preliminary data claims that combination therapy is positively associated with improvements in TGs and BP, but available data is extremely limited with only an overview of results being released by the manufacturer (Orexigen) [10, 62••].

Diabetes implication: Bupropion-zonisamide treatment has also been linked to decreased fasting insulin, but currently, there is not enough available research to confirm its cardiometabolic safety profile.

### Pramlintide + Metreleptin (Amylin/Leptin)

Pramlintide is an amylin synthetic analog that is hypothesized to influence amylin receptor activation in order to produce a satiating effect, reduce food intake and regulate short-term energy homeostasis [10, 63, 64]. Metreleptin is a leptin analog that has the potential to influence the hypothalamus in order to help regulate hunger cues and long-term energy homeostasis [10, 63, 64]. Thus far, there is a limited number of human trials evaluating the influence of amylin/leptin, although rodent trials indicate a synergistic effect that results in successful body weight reductions and improvements in cardiometabolic parameters [10, 64]. A human trial that looked at severe dietary restriction (up to 45 %) in addition to either combination therapy or monotherapy leads to a 11.5 kg, 12.7 %, reduction in initial body weight in the combination group in comparison to 7.4 kg (8.4 %) and 7.9 kg (8.2 %) reduction in the pramlintide and metreleptin monotherapy groups, respectively (Fig. 1) [63]. A phase 2 clinical trial in humans also documented a 9.2 % placebo-subtracted weight loss after 28 weeks of treatment [65]. The most common mild adverse events linked with amylin/leptin treatment are nausea and injection site irregularities [63].

Cardiometabolic implications: For patients with baseline normoglycaemic and lipidaemic measurements, amylin/leptin treatment resulted in decreased TC, LDL and TGs (Table 1) [63].

Diabetes implications: Amylin/leptin use has also been connected with beneficial influences on glycaemic markers such as FBG, insulin and HOMA-IR (Table 1) [63]. However, the two companies coordinating the preliminary trails for amylin/leptin completed a commercial assessment that ended in the decision to halt future research [64] which disallows any firm conclusions to be made in regard to the efficacy and cardiometabolic safety of this type of medication.

## Conclusion

In summary, the newer generation anti-obesogenic medications reviewed in this study frequently resulted in placebo-subtracted weight losses of approximately 3.0 to 9.0 kg. Although this may not seem like a large amount of weight loss, these results are substantially better than the average 2.0 to 3.0 kg of weight loss attained through 36 months of lifestyle intervention [18]. It is important to note that weight management medications are not linked with any permanent biological changes to mechanisms associated with obesity and as such do not generally have lasting weight loss effects after medicinal treatment has ceased [26]. This is problematic given that historically long-term (>2 years) adherence for weight management medications is minimal at approximately 2 % [3].

Newer generation anti-obesity agents have been associated with a multitude of cardioprotective effects and favourable glycaemic results which have lead to FDA approval of quite a few new obesity medications. Before patients begin pharmacological therapy, current health status, medication usage, medical history and regional approval all need to be considered. In concordance with the Endocrine Society, we recommend that for patients with a history of cardiovascular dysfunction or uncontrolled hypertension, non-sympathomimetic agents such as lorcaserin or orlistat be used as a first-line therapy in the pharmacological treatment of obesity. For patients with diabetes, GLP-1 agonists that have consistently documented positive glycaemic effects should be initially prescribed. Patients with both CVD and diabetes may also do well on liraglutide given its history of cardiac safety at lower doses in addition to the cardiometabolic improvements seen in recent literature. Overall, the beneficial cardiometabolic influences anti-obesogenic agents have largely not been assessed long term, with very little available data supporting reductions in long-term MACEs and mortality. In order to bridge these obvious gaps, heterogeneous longitudinal studies are required. Clinically, these results are promising, but as with any medication, the adverse events associated with their use must be kept in mind. Thus, when prescribing anti-obesogenic agents, health care providers should take care to properly monitor patients and cease medicinal treatment if the expected reductions in weight and cardiometabolic risk factors are not obtained within a reasonable period of time.
